# Exposure to cell phone radiations produces biochemical changes in worker honey bees

**DOI:** 10.4103/0971-6580.75869

**Published:** 2011

**Authors:** Neelima R. Kumar, Sonika Sangwan, Pooja Badotra

**Affiliations:** Department of Zoology, Punjab University, Chandigarh - 160 014, India

**Keywords:** *Apis mellifera*, biomolecules, cell phone radiations, hemolymph

## Abstract

The present study was carried out to find the effect of cell phone radiations on various biomolecules in the adult workers of *Apis mellifera* L. The results of the treated adults were analyzed and compared with the control. Radiation from the cell phone influences honey bees’ behavior and physiology. There was reduced motor activity of the worker bees on the comb initially, followed by en masse migration and movement toward “talk mode” cell phone. The initial quiet period was characterized by rise in concentration of biomolecules including proteins, carbohydrates and lipids, perhaps due to stimulation of body mechanism to fight the stressful condition created by the radiations. At later stages of exposure, there was a slight decline in the concentration of biomolecules probably because the body had adapted to the stimulus.

## INTRODUCTION

Cell phone usage is a major public health concern because of potential risk of chronic exposure to low level of radiofrequency and microwave radiation that pulse off the phone antennae in close proximity to the head.[1] These concerns have induced a large body of research, both epidemiological and experimental, in humans and animals. Honeybees are reliable indicators of environmental status and possess several important ecological, ethological, and morphological characteristics. They are the best experimental animals to study the effect of electromagnetic waves because they possess in their abdomen magnetite granules which help the bees in their orientation flight. Moreover, the integument of bees has semiconductor functions. It is in the light of these characteristics of honey bees that the present investigation was planned to study the metabolic changes with respect to proteins, carbohydrates, and lipids in hemolymph of worker honeybee of *Apis mellifera* L. exposed to cell phone radiation.

## MATERIALS AND METHODS

### Study area

The samples of *A. mellifera* adult worker bees were drawn from the colonies maintained by Department of Zoology, Punjab University, Chandigarh.

### Experimental design

A specially designed wooden box called the observation hive was used for the experiment. Front and back of the box were made up of glass while the two sides had wire gauze to ensure proper ventilation. Two such boxes, one experimental and the other control, were taken for the present study. The phones used were of the same make and model and had the same network. Phones were kept in listen-talk mode for 40 min using a tape recorder. Ten honeybees were collected from the exposed frame at intervals of 10, 20 and 40 min. Ten honeybees were collected from the control at the same time intervals.

### Sample preparation

Hemolymph of the worker bees was extracted with the help of the micropipette inserted into the inter-segmental region of the bee’s abdomen. Equal volume of hemolymph from all bees was dissolved in 1 ml of normal saline.

### Biochemical estimation

#### Estimation of total carbohydrates

The quantitative estimation of total carbohydrates in the test and control samples of *A. mellifera* was carried out by following the method of Sawhney and Singh.[2]

#### Estimation of glycogen

Seifter’s method[3] was followed for the estimation of glycogen in treated and non-treated hemolymph of *A. mellifera*.

#### Glucose estimation

For the estimation of glucose in the hemolymph of *A. mellifera*, the method of Somogyi and Nelson[4] was employed.

#### Estimation of total lipids

The quantitative estimation of total lipids in the treated and non-treated hemolymph extract of *A. mellifera* was carried out by following the procedure of Fringes and Dunn.[5]

#### Cholesterol estimation

Method of Zlatki’s *et al*.[6] was employed for the estimation of cholesterol in treated and non-treated sample.

#### Estimation of protein

The total quantity of protein in the test and control sample of *A. mellifera* was determined by following the standard procedure of Lowry.[7]

## RESULTS AND DISCUSSION

### Total carbohydrates

#### Control

In the non-treated or control sample, the concentration of total carbohydrates in the hemolymph was found to be 1.29±0.02 mg/ml.

#### Test

In the hemolymph of treated bees, the concentration was 1.5±0.04 mg/ml in 10 min, 1.73±0.01 mg/ml in 20 min and 1.61±0.02 mg/ml in 40 min exposure samples.

#### Glycogen

In the treated sample, the glycogen content (mg/ml) was found to be 0.019 0.001 as compared to 0.047 0.001, 0.076±0.001 and 0.028±0.002 in 10, 20 and 40 min exposure samples, respectively.

#### Glucose

The glucose content (mg/ml) in control sample was 0.218±0.0005, while in the various treated samples the concentration was 0.231±0.002, 0.277±0.001 and 0.246±0.002 in 10, 20 and 40 min exposure samples, respectively.

#### Total lipids

The concentration of total lipids (mg/ml) in the hemolymph of control worker bee was found to be 2.06±0.02. For the treated sample, the concentration of total lipids was 3.03±0.02, 4.50±0.035 and 3.10±0.02 in 10, 20 and 40 min exposure samples, respectively.

#### Cholesterol

The cholesterol concentration (mg/ml) in the non-treated sample was 0.230 0.001. In the treated sample, the concentration was 1.381±0.002, 2.565±0.002 and 1.578±0.002 in 10, 20 and 40 min exposure samples, respectively.

#### Total protein content

In the hemolymph of control sample, the protein concentration (mg/ml) was 0.475±0.002. In the treated sample, the protein concentration was 0.525±0.003, 0.825±0.0001 and 0.650±0.0003 in 10, 20 and 40 min exposed samples, respectively.

Very little work has been done on biochemical, metabolic and physiological influences of cell phone radiations pertaining to health risk in man.[8] Therefore, the present investigations on the influence of cell phone radiations on some biochemical and physiological aspects of honeybee biology were undertaken. That the behavior of honeybee is altered to some extent by high or low energy fields or electromagnetic radiations has been known for quite some time.[9]

During the present investigation, it was observed that there was an increase in concentration of total carbohydrates in the bees exposed to cell phone radiation for 10 min as compared to unexposed or control bees. Increasing the exposure time to 20 min resulted in further increase in the concentration, while an exposure of 40 min had a reverse effect and there was a decline in carbohydrate concentration, though it was still higher as compared to control. Hemolymph glycogen and glucose content also showed the same trend, i.e., there was increase in content up to 20 min exposure after which there was a slight decline in the concentration which remained more than the control. Sharma[10] had also reported increase in glycogen and glucose levels in the exposed pupa of *A. mellifera*.

Lipids are the major energy reserves of insects. Certain lipid classes are structure components of membranes while others are raw materials for a variety of hormones and pheromones. Estimation of total lipids and cholesterol during the present study showed that the trend was similar to that of carbohydrates. After an initial increase in concentration at the 10 and 20 min exposure period, a decline was observed in the concentration of total lipids and cholesterol at 40 min exposure.

It was interesting to note that during the present study as the exposure time increased, it appeared that the bees having assessed the source of the disturbance decided to move and a large scale movement of the workers toward the talk-mode (not toward the listening mobile) was observed. Also, the bees became slightly aggressive and started beating their wings in agitation. This mobility of the bees could be responsible for increase utilization of energy sources and consequent decrease in concentration of carbohydrates and lipids in the 40 min exposed sample.

## References

[1] Carlo G Radiation is killing the bees, despite the cell phone industry’s disformation. http://buergerwelle.de/pdf/radiation-is-killing-the-bees.htm.

[2] Sawhney SK, Singh R (2000). Estimation of total carbohydrates by anthrone reagent method. Introductory practical Biochemistry.

[3] Seifter S, Seymour S, Novic E, Muntwyler E (1950). Determination of glycogen with anthrone reagent.Vol.

[4] Somogyi M, Nelson N (1945). A photometric adaptation of the Somogyi method for the determination of glucose. J Biol Chem.

[5] Fringes CS, Dunn RT (1970). A colorimetric method for determination of total serum lipids based on the sulphophospho-vanillin reaction. American J Clin Pathol.

[6] Zlatki’s A, Zak B, Boyle AJ (1953). A new method for direct determination of serum cholesterol. J Lab Clin Med.

[7] Lowry OH, Rosenbrough NJ, Farr AI, Randall RJ (1951). Protein measurement with the folin phenol reagent. J Biol Chem.

[8] Boscol P, Di Sciascio MB, D’ Ostillo S, Del Signore A, Reale M, Conti P (2001). Effects of electromagnetic fields produced by radio television broadcasting stations on the immune system of women. Sci Total Environ.

[9] Cartensen EL (1987). Biological effects of transmission line fields. Bioelectromagnetics.

[10] Sharma A (2008). Biochemical changes in *Apis mellifera* L. Worker Brood Induced by Cell Phone Radiation. M Phil. Thesis, Department of Zoology.

